# THE MODERATING EFFECT OF RAISING ONE’S GRANDCHILDREN ON THE RELATION BETWEEN SLEEP AND DEPRESSION

**DOI:** 10.1093/geroni/igz038.2505

**Published:** 2019-11-08

**Authors:** Melanie Stearns, Danielle K Nadorff

**Affiliations:** Mississippi State University, Starkville, Mississippi, United States

## Abstract

Recent evidence has shown that poor quality sleep is associated with depression, particularly among older individuals (Bao et al., 2017; Nadorff, Fiske, Sperry, & Petts, 2012). Moreover, given the high prevalence of depressive symptoms among older adults, it is important to identify possible risk factors of poor sleep quality. One possible risk factor is being a custodial grandparent (raising one’s grandchildren), as increased caregiving responsivities are associated with increased depressive symptoms (Brand-Winterstein, Edelstein, & Bachner, 2018). Based upon these previous findings, the current study examines the effect of custodial status on the relation between sleep quality and depressive symptoms. The sample (N = 466) was a subset of individuals recruited in the second wave of the MIDUS biomarkers project completed in 2009 who answered the sleep, caregiving, and depressive symptoms variables of interest. Measures included the Center for Epidemiological Studies Depression Scale (CESD), the Pittsburgh Sleep Quality Index (PSQI), and a question regarding custodial grandparent status. The current study aimed to examine whether poor sleep quality might serve as a risk factor for experiencing depressive symptoms and how custodial grandparents might differ from other older adults. Moderation analyses were conducted using SPSS’ Process macro on the sample. The interaction between global sleep quality and custodial grandparent status was significant in predicting depressive symptoms, t (1, 465) = 3.90, p = .04, such that custodial grandparents reported a stronger positive correlation between greater global sleep problems and depressive symptoms than non-custodial grandparents. Implications, future directions, and limitations are discussed.

